# *Ganoderma lucidum* as a Functional Bioactive Candidate for Glycemic Regulation: Mechanisms, Preclinical Evidence, and Clinical Translation

**DOI:** 10.3390/metabo16050334

**Published:** 2026-05-15

**Authors:** Bogdan Florea, Doru Morar, Corina Marina Kracunovic, Simina Velescu, Vlad Iorgoni, Paula Nistor, Janos Degi, Ionica Iancu, Maria-Larisa Ardelean (Rusu), Romeo Teodor Cristina, Alexandra Pocinoc, Eugenia Dumitrescu

**Affiliations:** 1 Department of Internal Medicine, University of Life Sciences “King Mihai I” from Timisoara, 300645 Timisoara, Romania; bogdan-alexandru.florea.fmv@usvt.ro (B.F.); dorumorar@usvt.ro (D.M.); corina.kracunovic@usvt.ro (C.M.K.); simina.velescu@usvt.ro (S.V.); 2 Doctoral School “Veterinary Medicine”, University of Life Sciences “King Mihai I” from Timişoara, Calea Aradului 119, 300645 Timişoara, Romania; vlad.iorgoni@usvt.ro (V.I.); paula.nistor@usvt.ro (P.N.); maria-larisa.ardelean.fmv@usvt.ro (M.-L.A.); alexandra.pocinoc@usvt.ro (A.P.); 3 Department of Infectious Diseases and Preventive Medicine, University of Life Sciences “King Mihai I” from Timisoara, 300645 Timisoara, Romania; janosdegi@usvt.ro (J.D.); ionica.iancu@usvt.ro (I.I.); 4 Department of Pharmacy and Pharmacology, University of Life Sciences “King Mihai I” from Timisoara, 300645 Timisoara, Romania; eugeniadumitrescu@usvt.ro; 5 Department of Parasitology, University of Life Sciences “King Mihai I” from Timisoara, 300645 Timisoara, Romania

**Keywords:** *Ganoderma lucidum*, functional food, bioactive compounds, type 2 diabetes, metabolic syndrome, insulin resistance, polysaccharides, triterpenoids, gut microbiota, glycemic control

## Abstract

Type 2 diabetes mellitus (T2DM) is a major global health challenge that has intensified interest in multi-target nutraceuticals with potential adjunctive benefits. *Ganoderma lucidum* (Lingzhi/Reishi) is a medicinal mushroom traditionally used in East Asia and is increasingly investigated for its role in glycemic regulation and metabolic disturbances. This review critically synthesizes current evidence on its hypoglycemic effects, focusing on bioactive compounds, molecular mechanisms, and translational limitations. Unlike broader reviews on Ganoderma bioactivity and health-related benefits, this review specifically evaluates the alignment between taxonomic authentication, chemical standardization, preclinical mechanisms, and human clinical evidence in the context of glycemic regulation. This narrative review was based on a targeted literature search conducted in PubMed/MEDLINE, Web of Science, and Scopus for studies published up to October 2025, supplemented by Google Scholar. The included studies comprised in vitro experiments, in vivo animal models, and human clinical trials evaluating glycemic and metabolic outcomes of Ganoderma preparations. In vitro and animal studies indicate that polysaccharides, including β-(1→3)/(1→6)-glucans and proteoglycans such as FYGL, may improve insulin sensitivity via AMPK (AMP-activated protein kinase) and PI3K/Akt pathways, promote GLUT4 (glucose transporter type 4) translocation, suppress hepatic gluconeogenesis, protect pancreatic β-cells, and modulate gut microbiota. In enzyme assays and preclinical models, lanostane-type triterpenoids act primarily by inhibiting α-glucosidase and α-amylase, thereby potentially reducing postprandial glucose excursions. Despite consistent preclinical evidence, clinical findings remain heterogeneous, with the largest randomized controlled trial reporting no significant glycemic benefit. Overall, *Ganoderma lucidum* shows strong mechanistic plausibility but insufficient clinical evidence for antidiabetic efficacy. Future research should prioritize species authentication, chemical standardization, and adequately powered clinical trials.

## 1. Introduction

The increasing worldwide prevalence of type 2 diabetes mellitus (T2DM) has further propelled interest in the search for safe and multi-target nutraceuticals as potential adjuncts to existing treatments. *Ganoderma lucidum* (GL), a polypore fungus in the family of basidiomycetes, has been used in traditional medicines in the ancient East for many centuries [1]. Over the past two decades, advances in phytochemistry and pharmacological research have clarified the major bioactive constituents of *G. lucidum*, primarily water-soluble polysaccharides and lanostane-type triterpenoids [2,3,4].

In this review, the name *Ganoderma lucidum* is used in the strict European sense (sensu stricto) while acknowledging that many commercially cultivated “Lingzhi” materials in East Asia correspond to *Ganoderma sichuanense* rather than *G. lucidum* sensu stricto.

There appears to be an accumulating body of evidence for the complementary metabolic actions of these components in experimental diabetes models. Lanostane-type triterpenoids have been linked mainly to inhibition of intestinal carbohydrate-digesting enzymes, including α-glucosidase and α-amylase [5,6,7,8]. Ganoderma polysaccharides and proteoglycan fractions have been associated with improved insulin signaling, peripheral glucose uptake, AMPK activation, and modulation of hepatic glucose output [9,10,11,12,13]. Additional preclinical studies suggest β-cell protective, antioxidant, anti-inflammatory, and microbiota-related effects [14,15,16,17,18]. Collectively, these findings support the biological plausibility of *G. lucidum* as a multi-target modulator of glucose homeostasis. Within this framework, *G. lucidum* may be regarded as a functional food-derived source of bioactive compounds with potential relevance to hyperglycemia, insulin resistance, oxidative stress, intestinal dysbiosis, and broader metabolic dysfunction [19,20].

Although several recent reviews have addressed the broad chemistry, bioactivity, antioxidant effects, safety, and health-related applications of Ganoderma lucidum and related Ganoderma species, including the recent review by Plosca et al. in *Antioxidants*, the present review differs in both scope and translational emphasis. Previous reviews have generally discussed *G. lucidum* as a multifunctional medicinal mushroom across a wide spectrum of biological activities, whereas the present manuscript focuses specifically on glycemic regulation and metabolic translation. In particular, this review critically aligns taxonomic authentication, tissue source, extraction method, polysaccharide and triterpenoid standardization, mechanism-level evidence, and clinical trial limitations within a single glycemic regulation framework. Another distinctive feature is the explicit separation of evidence derived from in vitro assays, animal models, and human clinical studies, in order to avoid direct extrapolation from preclinical mechanisms to clinical efficacy. Therefore, the novelty of this review lies not in presenting *G. lucidum* as a newly discovered bioactive organism but in evaluating whether its mechanistic plausibility can be translated into clinically meaningful glycemic outcomes under conditions of rigorous species authentication and chemical standardization [21].

### Literature Search and Selection Approach

This study is a narrative review based on a targeted and structured literature search. The search was designed to identify experimental, preclinical, and clinical studies relevant to Ganoderma-derived bioactive compounds and glycemic regulation.

A literature search was conducted in PubMed/MEDLINE, Web of Science Core Collection, and Scopus for studies published up to 31 October 2025. Additional sources were identified through Google Scholar and reference list screening for supplementary citation tracking. The last search was performed on 2 November 2025.

The search strategy used combinations of controlled vocabulary and free-text terms related to the fungal material, bioactive compounds, mechanisms, and metabolic outcomes. Representative search terms included: “Ganoderma lucidum”, “Lingzhi”, “Reishi”, “Ganoderma polysaccharides”, “β-glucans”, “proteoglycan”, “FYGL”, “triterpenoids”, “ganoderic acid”, “ganoderol”, “diabetes”, “type 2 diabetes”, “hyperglycemia”, “insulin resistance”, “glycemic control”, “fasting glucose”, “HbA1c”, “HOMA-IR”, “oral glucose tolerance test”, “AMPK”, “PI3K/Akt”, “GLUT4”, “gluconeogenesis”, “α-glucosidase”, “α-amylase”, “gut microbiota”, and “clinical trial”. Boolean combinations using AND/OR were adapted to each database.

Studies were considered eligible if they met the following inclusion criteria: (i) investigated Ganoderma lucidum, Ganoderma-derived preparations, or clearly related Ganoderma bioactive fractions; (ii) evaluated glycemic, metabolic, or mechanistic outcomes relevant to glucose homeostasis; (iii) including in vitro experiments, in vivo animal models, or human clinical studies; and/or (iv) reporting information on bioactive compounds, tissue source, extraction method, species authentication, or chemical characterization relevant to interpretation of the metabolic findings.

Studies were excluded if they: (i) focused exclusively on non-metabolic outcomes without relevance to glycemic regulation; (ii) evaluated mushroom products without sufficient identification of Ganoderma as the studied material; (iii) were conference abstracts, editorials, patents, or non-peer-reviewed sources without sufficient methodological details; (iv) lacked accessible full text; or (v) duplicated data already reported in a more complete publication.

The screening process was performed in stages. First, titles and abstracts were screened for relevance to Ganoderma bioactivity and metabolic or glycemic outcomes. Second, potentially relevant full-text articles were assessed for eligibility according to the inclusion and exclusion criteria. Third, eligible studies were grouped according to evidence type: in vitro mechanistic studies, animal studies, human clinical studies, and review or methodological papers. Greater emphasis was placed on studies that clearly reported species identity, fungal tissue source, extraction method, chemical standardization, dose, duration, and glycemic endpoints such as fasting glucose, HbA1c, HOMA-IR (homeostatic model assessment of insulin resistance), insulin levels, oral glucose tolerance, or mechanistic pathways involved in glucose homeostasis.

Because this manuscript is a narrative review rather than a systematic review, no formal meta-analysis was performed. However, the origin of evidence was considered during interpretation, and conclusions were weighted according to study design, translational relevance, and methodological robustness.

For mechanistic and model-specific claims, priority was given to primary experimental studies. Review articles were used mainly to contextualize broader trends, summarize the field, or support general background statements, but not as the principal evidence for specific molecular, cellular, or animal model findings.

## 2. Taxonomy, Nomenclature, and Authentication

The currently accepted scientific name is *Ganoderma lucidum* (Curtis) P. Karst. According to MycoBank and Index Fungorum, it is classified in Fungi, *Basidiomycota*, *Agaricomycetes*, *Polyporales*. The species was originally described as *Boletus lucidus* by Curtis in 1781 and later transferred to the genus *Ganoderma* by P.A. Karsten in 1881. The currently accepted name is *G. lucidum* (Curtis) P. Karst., as listed in MycoBank and Index Fungorum [22,23]. As the type species of the genus, *G. lucidum* has played a central role in defining the European concept of the laccate, or “varnished”, Ganoderma group [24,25].

### 2.1. Nomenclatural Background and Typification

**Figure 1:**
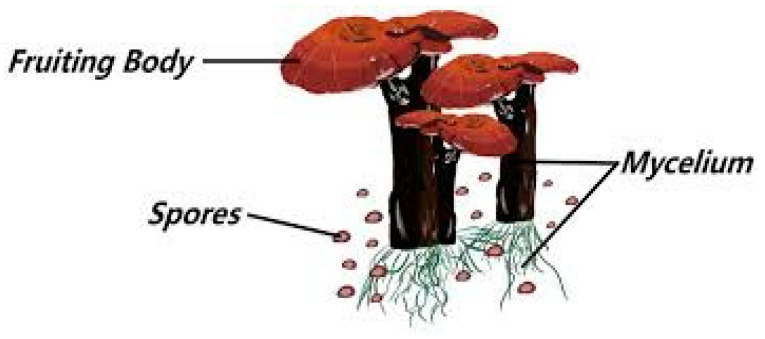
Morphological aspect of *Ganoderma lucidum* [26,29].

This botanical species combination of *G. lucidum*, as described by Karsten, was derived from Curtis’s earlier taxonomic treatment of Boletus lucidus [26,27]. Essentially, both taxa are responsible for the nomenclatural basis of the European laccate Ganoderma species. Designating *G. lucidum* as the type species has contributed significantly to its long-term taxonomic classification (Figure 1) [25,28].

Some infraspecific taxa, such as *G. lucidum* var. *badium* and var. *typicum*, have been described in the older literature. However, such names have not been currently in use in modern classifications and have been abandoned in favor of species-level taxa based on molecular data [30].

### 2.2. The “Lingzhi–Reishi” Complex and Frequent Misapplication

Although the terms “Lingzhi” and “Reishi” are often used interchangeably with *G. lucidum*, molecular phylogenetic studies have shown that many cultivated Asian strains correspond to *Ganoderma sichuanense* rather than *G. lucidum* sensu stricto. Indeed, multigene analyses using the ITS, tef1, and rpb2 datasets have constantly shown that most Asian strains belong to *G. sichuanense*. After reevaluation of the *G. lingzhi* type specimens, *G. lingzhi* is today recognized as a later synonym of *G. sichuanense* [31,32,33].

In contrast, *G. lucidum* sensu stricto is mainly distributed in the European and North Temperate region and most commonly associated with hardwood hosts. The difference between the two species is not merely a matter of classification but also has implications regarding phytochemical and translational research, since triterpenoids and polysaccharides can mostly be linked to species lineage. For example, European *G. lucidum* sensu stricto and cultivated Asian Lingzhi materials now frequently assigned to G. sichuanense may differ in their lanostane-type triterpenoid fingerprints, including the relative abundance of ganoderic acids, ganoderenic acids, ganoderols, and related oxidized lanostane derivatives. Such differences can influence α-glucosidase inhibitory activity, antioxidant potential, and other bioactivities attributed to organic extracts. Similarly, polysaccharide yield, branching pattern, molecular-weight distribution, and protein conjugation may vary according to species identity, substrate, developmental stage, and cultivation system. Therefore, studies using materials labeled only “*G. lucidum*” without molecular authentication may not be directly comparable, especially when the biological activity is linked to defined triterpenoid or polysaccharide profiles. Reproducible research, especially regarding biological phenomena, is dependent upon accurate identification of species [34,35].

From a translational perspective, materials labeled “*G. lucidum*” and produced in East Asia should be taxonomically verified, because widely cultivated Chinese Lingzhi corresponds to *G. sichuanense* rather than *G. lucidum* sensu stricto. Accurate species assignment improves comparability across pharmacological and clinical studies [27,36].

### 2.3. Morphological Diagnosis of G. lucidum

Morphologically, *G. lucidum* typically forms annual or too-short-lived perennial basidiocarps that may be stipitate or sessile and usually exhibit a laccate, reddish-brown- to chestnut-colored pileus with concentric zoning. The context is corky to woody, and the pore surface is composed of small, rounded pores. Microscopically, the species is characterized by a trimitic hyphal system and broadly ellipsoid, double-walled basidiospores with a truncate apex [34].

Amongst the laccate Ganoderma species, there is often confusion between *G. tsugae*, found in North America, and *G. multipileum* in tropical and subtropical Asia. However, host preference, geographical distribution, and multilocus DNA analysis are needed for accurate differentiation. It is, however, pertinent to note that most Ganoderma species sold as “Lingzhi” in East Asia belong to *G. sichuanense*, not *G. lucidum* sensu stricto, which has implications for pharmacological analysis [34,37].

**Figure 2:**
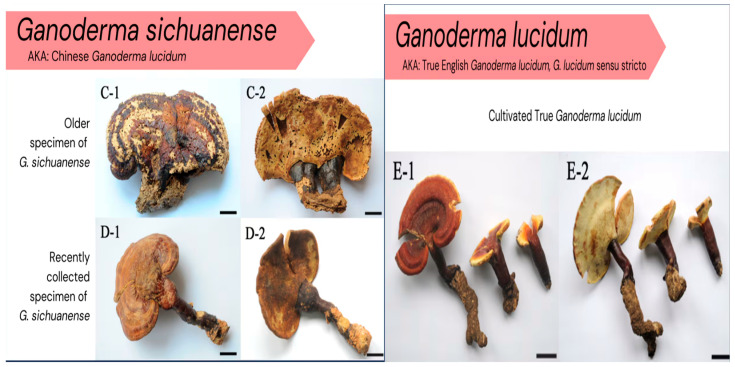
Representative macroscopic morphology of *Ganoderma lucidum* sensu stricto and *Ganoderma sichuanense*. Both species may show laccate, reddish-brown basidiocarps with overlapping morphological features; therefore, visual inspection alone is insufficient for species-level authentication and should be complemented by DNA-based identification [29,36].

The representative images shown in Figure 2 are intended to support visual comparison only. Because laccate Ganoderma species may show overlapping basidiocarp morphology, photographic comparison should be considered illustrative rather than diagnostic. Reliable discrimination between closely related species requires molecular authentication, preferably using ITS sequencing supplemented by tef1 and rpb2 markers [34,36,38].

### 2.4. Ecology, Substrate Preference, and Biogeography

*G. lucidum* sensu stricto is primarily saprotrophic, although it may occasionally exhibit weak parasitic behavior on hardwood hosts. Its verified geographical distribution is restricted mainly to Europe and temperate Asia, while authentic reports in North America are few and may refer instead to related species, especially *G. tsugae* on hemlock, Tsuga [37,39]. Correspondingly, most commercial “Lingzhi” in Asia is represented by *G. sichuanense*, which is usually produced on sawdust media, hardwood logs, or bag culture. Many environmental and biotechnological factors, such as substrate composition, temperature, moisture, and aeration, play important roles in regulating mycelial growth, fruiting body shape, and secondary metabolite production [40,41,42].

### 2.5. Chemosystematics Considerations

All laccate Ganoderma species produce characteristic lanostane-type triterpenoids and β-glucan-rich polysaccharides. Nevertheless, their proportional production and microscopic details are dependent on evolutionary lineage and habitat. More recently, there have been observations that such chemical traits may show substantial concordance with species-level taxonomic identity within laccate Ganoderma [5,34]. Therefore, species-specific verification, rather than common taxonomy as “*G. lucidum*,” is critical to effective pharmacological assessment. Moreover, incorporating species-specific verification with targeted metabolite profiling helps to create a robust platform for chemotaxonomic analysis and improves the accuracy of comparative and translational research [41,43,44].

### 2.6. DNA Barcoding and Authentication Process

To ensure that biological and pharmacological observations are properly associated with a confirmed taxon, a minimum authentication system is proposed. Specimens should be provided to an official public herbarium or culture collection and numbered to facilitate tracing [31,45]. DNA barcode sequencing should involve Sanger sequencing of the ITS region as the core barcode, supplemented by tef1 and rpb2 markers to resolve relationships within laccate Ganoderma better. For those studies that allow it, supplying GenBank accessions and phylogenetic topologies, including type-derived sequences, is essential [38,46,47].

The identity should then be verified against meticulously curated databases such as MycoBank and Index Fungorum, with a clear point as to whether this relates to *G. lucidum* sensu stricto or *G. sichuanense*. Furthermore, it is a major advantage if there is complete openness as to whether the biological material relates to the fruiting body, a mycelium culture, or a spore-derived product, as well as details as to cultivation conditions and geographical origin [25,48]. Lastly, profiling for characteristic metabolites such as specified lanostane triterpenoids, combined with polysaccharide characteristics, needs to be integrated with genetic information to provide robust verification support [34].

## 3. Phytochemical Composition Relevant to Glycemic Control

The major Ganoderma-derived compound groups most relevant to glycemic regulation are water-soluble polysaccharides, especially β-(1→3)/(1→6)-glucans and proteoglycan fractions such as FYGL, and lipophilic lanostane-type triterpenoids, including ganoderic acids, ganoderenic acids, ganoderols, and related derivatives. These two groups act through complementary mechanisms. GLPs are mainly associated with insulin sensitization, AMPK activation, IRS1–PI3K–Akt signaling, GLUT4 translocation, and hepatic gluconeogenesis suppression [9,10,11,12,13]. They have also been linked to pancreatic β-cell protection and gut microbiota modulation in preclinical studies [11,14,15,16,18]. In contrast, lanostane-type triterpenoids contribute primarily through intestinal carbohydrate-digesting enzyme inhibition, especially α-glucosidase and, in some models, α-amylase inhibition, thereby potentially reducing postprandial glucose excursions [5,7,8]. Spore-derived preparations may also contain triterpenoid-rich lipid fractions and polysaccharide components, but their metabolic effects are more dependent on processing method, sporoderm disruption, and chemical standardization [20,49].

The principal phytochemical classes relevant to glycemic regulation and their proposed metabolic effects are summarized in Table 1.

The information summarized in Table 1 is based on representative phytochemical, mechanistic, and review evidence on Ganoderma polysaccharides, proteoglycans, triterpenoids, phenolic compounds, and spore-derived fractions [1,2,5,7,11,13,21,43,49,50,51,52,53,54].

### 3.1. Polysaccharides (GLPs)

The water-soluble polysaccharides of *G. lucidum* comprise structurally diverse β-glucans, heteropolysaccharides, glycoproteins, and proteoglycans. These include β-glucans, in which the backbone is defined by β (1→3) bonds and is further substituted at the 6-position by β (1→6) bonds and any number of proteoglycans and glycoproteins. Of these, the FYGL polysaccharide (Fudan–Yueyang–*G. lucidum*) has been well studied and has been shown to be defined by a protein backbone and an attached polysaccharide moiety [50,51].

Reported molecular-weight distribution ranges very widely, from around 10 kDa to more than 1 MDa. The level of both branching and protein conjugation has been found to affect biological activity. Analytical analysis would include total carbohydrate determination by the phenol sulfuric acid assay, molecular-weight and polydispersity analysis by HPSEC-MALS, monosaccharide analysis by PMP-HPLC or GC/MS of alditol acetates, and linkage analysis by methylation analysis and GC/MS. The confirmation of the structure of the β(1→3)/(1→6) glycosidic linkages would typically be accomplished by FTIR and ^1^H/^13^C NMR spectroscopy [38,50,55].

#### 3.1.1. Mechanistic Relevance to Glucose Homeostasis

GLPs exert complementary multi-target effects on glucose metabolism. At the intestinal level, some β-glucans and polysaccharide fractions display moderate α-glucosidase inhibitory activity, leading to diminished postprandial glycemia [51,56,57]. At the insulin-sensitive tissue level, *G. lucidum* polysaccharides have been reported to enhance insulin signaling, including PI3K/Akt-related pathways, and to promote GLUT4 expression or translocation in experimental models [9,10,11,12,58]. AMPK activation has also been described in obese and diabetic animal models treated with Ganoderma preparations [9,59].

The hepatic actions include inhibition of key gluconeogenic enzymes, particularly phosphoenolpyruvate carboxykinase and glucose-6-phosphatase, causing decreased endogenous glucose output [10,12,13,60]. Moreover, GLPs have been reported to preserve pancreatic β-cells by reducing oxidative and inflammatory injury in diabetic models [11,13,16,58]. Separately, Ganoderma polysaccharides may display prebiotic-like properties by regulating intestinal microbiota, enhancing short-chain fatty acid-producing taxa, and improving epithelial barrier function [14,15,18]. The proteoglycan FYGL shows additional α-glucosidase inhibition as a function of dosage and ameliorates insulin resistance and fasting glucose in diabetic models by interacting with insulin signaling and glucose output from the liver [11,13,51].

#### 3.1.2. Structure–Activity Considerations

Structure–activity analysis has indicated an optimal range of molecular weight and higher degrees of branching for polysaccharides, and this has led to higher levels of immunometabolic activity. Protein conjugation, as seen in proteoglycans such as FYGL, could be particularly effective in this regard by promoting interaction with recognition receptors and subsequent signal transduction in metabolic processes. These characteristics are in keeping with those reports suggesting an improvement in glycemic control in preclinical evaluations of natural polysaccharides as potential antidiabetic agents [14,41,54].

### 3.2. Lanostane-Type Triterpenoids

Ganoderma species produce various lanostane-type triterpenoids, including ganoderic acids, ganoderenic acids, ganoderols, and ganodermanols. The structural variations in these compounds result from the differences in the degree of oxidation of some positions, like C3, C7, C11, C15, C23, and C26, and the termination of the side chain by carboxylic acid, aldehyde, or alcoholic groups. Because of their lipophilic properties, these compounds can be found mostly in organic solvent extracts like ethyl acetate, methanol, or chloroform. HPLC or UPLC Diode Array Detectors or LC MS/MS analysis is mostly used for quantifying the compounds, while higher structural information can be obtained through LC MS/MS analysis [5,52].

#### 3.2.1. Enzyme Inhibition and Postprandial Glucose Control

Ganoderol B is frequently cited as a representative lanostane-type triterpenoid with α-glucosidase inhibitory activity; in one study, it showed an IC_50_ of approximately 120 μM in a yeast α-glucosidase assay. However, comparisons across studies and with acarbose should be interpreted cautiously because assay conditions vary [5,7].

Structure–activity relationship analyses have made it clear that the presence of a βhydroxyl group at position C3 and an unchanged carboxyl functional group in the side chain enhances inhibitory activity, which decreases by esterification. However, smaller degrees of unsaturation are preferable. These results can account for the varying degrees of inhibition on the enzyme found among different Ganoderma extracts [5,8].

#### 3.2.2. In Vivo Hypoglycemic Evidence

Enriched or purified triterpenoid derivatives have been shown to have hypoglycemic effects in diabetic animal models, with reduced fasting blood glucose levels and/or improved OGTT (oral glucose tolerance test) [61]. Many compounds have also been found to have anti-inflammatory or antioxidant activity, although their role in glycemia appears more directly associated with inhibiting enzymes that degrade carbohydrates, as well as secondary actions involving insulin action and/or lipids [5,17,21]. There have been numerous natural product surveys published over the past several decades that provide a compendium of these triterpenoid compounds, as well as their respective analytical and pharmacological data [52].

### 3.3. Stability, Bioavailability, and Additional Phytochemicals

The biological relevance of Ganoderma-derived phytochemicals depends not only on their abundance in the raw material but also on their stability during processing, gastrointestinal digestion, and absorption. Water-soluble polysaccharides are generally resistant to digestion by human enzymes in the upper gastrointestinal tract and may reach the colon, where they can be fermented by gut microbiota [14,15,18]. Their metabolic effects may therefore depend partly on molecular weight, branching degree, solubility, fermentability, and the production of short-chain fatty acids [43,54]. However, high-molecular-weight polysaccharides usually have limited direct systemic absorption, suggesting that many of their effects may be mediated through intestinal, microbiota-related, or immunometabolic mechanisms rather than through high circulating concentrations of intact polysaccharides [14,18].

Lanostane-type triterpenoids, including ganoderic acids, ganoderenic acids, ganoderols, and ganodermanols, are more lipophilic and may have limited aqueous solubility and variable oral bioavailability. Ganoderic acids are among the most characteristic and widely studied triterpenoids of Ganoderma species and should be considered key marker compounds for chemical standardization. Their stability and recovery may be influenced by extraction solvent, drying temperature, storage conditions, and formulation strategy. Because triterpenoids are commonly enriched in ethanolic, methanolic, chloroform, ethyl acetate, or spore-derived lipid extracts, direct comparison of studies requires a detailed reporting of extraction conditions and marker-compound quantification [5,21,52,62].

Phenolic compounds also contribute to the phytochemical profile of *G. lucidum*, although they are usually discussed less prominently than polysaccharides and triterpenoids. These compounds may include phenolic acids and other antioxidant constituents that contribute to free-radical scavenging, metal-chelating capacity, and inhibition of lipid peroxidation. Their relevance to glycemic regulation is likely indirect, through attenuation of oxidative stress and inflammatory signaling that contribute to insulin resistance and β-cell dysfunction. Nevertheless, most phenolic-related evidence remains based on in vitro antioxidant assays or animal models, and their independent contribution to clinical glycemic outcomes remains uncertain [2,21,53,62,63,64].

### 3.4. Tissue-Specific Profiling: Fruiting Bodies, Mycelium, and Spores

#### 3.4.1. Quantitative Variations in Polysaccharide and Triterpenoid Substances

Depending on different tissue types and processing parameters, phytochemical content in *G. lucidum* fruiting bodies can vary greatly [65]. For example, fruiting bodies can be considered an equal source of both hydrophilic and lipophilic phytoconstituents [53]. Moreover, polysaccharides can be effectively extracted by hot water, while lanostane triterpenoids can be concentrated in ethanolic and ethyl acetate extracts. Mycelium cultured through submerged/solid-state fermentation can be effectively optimized for polysaccharide content, while exo- and endoβ-glucans isolated through bioreactor culture exhibit α-glucosidase inhibitory activity [66].

Extraction methodology is another important determinant of phytochemical yield and composition. In addition to conventional hot-water extraction for polysaccharides and organic solvent extraction for triterpenoids, greener and intensified extraction approaches have increasingly been applied to Ganoderma materials. These include ultrasound-assisted extraction, microwave-assisted extraction, enzyme-assisted extraction, pressurized liquid extraction, and deep eutectic solvent-based methods. Such techniques may improve extraction efficiency, reduce solvent consumption, shorten extraction time, and enhance recovery of polysaccharides, triterpenoids, phenolics, or antioxidant fractions. However, extraction intensity, temperature, solvent polarity, pH, and processing duration may also alter molecular-weight distribution, branching structure, protein conjugation, or triterpenoid stability. Therefore, the extraction method should be reported in detail and considered when comparing the phytochemical composition and biological activity of different Ganoderma preparations [43,54,65,66,67].

Spores and their products, whether sporoderm-broken or sporoderm-removed powders, as well as spore oils, show specific lipid contents, triterpenoids, and simultaneously, water-soluble immunometabolic polysaccharides [49]. In biological assays, the effect of the preparation made from spores has been observed as a lowering in blood glucose concentrations in diabetic models, of 20% on average, for preparations administered at 300 mg·kg^−1^ on prolonged treatment courses, but the chemical composition and its biological effect depend dramatically on sporoderm preparation [20].

#### 3.4.2. Translation into Clinical Application

Owing to the delicacy of the polysaccharide/terpenoid ratio and structure of GLPs, standardized approaches are necessary for consistency and relevance to therapies directly addressing glycemia. On this note, it would be highly desirable for all experimental analyses assessing glycemia as endpoints to include specification for tissue origin and processing techniques and employ a two-way chemical signature approach incorporating both polysaccharide and representative triterpenoid compounds within their respective quantitative assessments for comparative purposes [1,43,67]. Adding a minimum of a functionally relevant assay, perhaps for α-glucosidase inhibition, AMPK activation, or GLUT4 cellular translocation, would enhance compatibility and allow for broader comparative analyses with future investigations as it pertains to translations (Table 2) [8].

A detailed analytical and methodological standardization framework is provided in Supplementary Table S1 [1,2,5,7,11,13,43,49,50,51,52,54].

## 4. Mechanistic Basis of Glycemic Modulation by *G. lucidum*

### 4.1. Intestinal Enzyme Inhibition and Postprandial Glucose Control

Lanostane-type triterpenoids isolated from Ganoderma, including ganoderol B and several ganoderic and ganoderenic acids, have been shown to inhibit intestinal carbohydrate-digesting enzymes, most notably α-glucosidase and, in some experimental settings, α-amylase. By slowing the hydrolysis of dietary carbohydrates, these compounds reduce the rate of glucose absorption and attenuate postprandial glycemic excursions, an effect conceptually comparable to that of the clinical α-glucosidase inhibitor acarbose [5,7,8].

**Figure 3:**
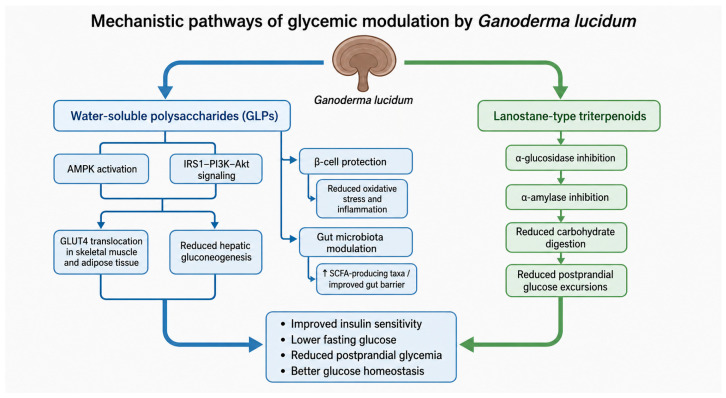
Mechanistic pathways underlying the potential glycemic regulatory effects of *G. lucidum*. Water-soluble polysaccharides and proteoglycan fractions have been reported in experimental models to modulate complementary insulin-related and energy-sensing pathways. IRS1–PI3K–Akt signaling is associated with insulin-dependent GLUT4 translocation, whereas AMPK activation contributes to energy sensing, glucose uptake, fatty acid oxidation, and suppression of hepatic glucose output. These pathways should be interpreted as parallel or convergent mechanisms rather than as a direct causal sequence. Additional preclinical mechanisms include suppression of hepatic gluconeogenesis; protection of pancreatic β-cells through reduced oxidative stress, inflammatory signaling, and apoptosis-related pathways; and modulation of gut microbiota with increased short-chain fatty acid production, improved epithelial barrier integrity, and reduced metabolic endotoxemia. Lanostane-type triterpenoids act mainly at the intestinal level by inhibiting α-glucosidase and α-amylase, thereby potentially attenuating postprandial glucose excursions. The mechanisms summarized in this figure are based mainly on in vitro and animal evidence and should not be interpreted as confirmed clinical efficacy in humans.

Among these constituents, ganoderol B exhibits sub-millimolar inhibitory potency in standard yeast α-glucosidase assays, with reported IC_50_ values around 120 μM, exceeding the activity of acarbose under identical assay conditions [7]. Related ganoderic acid derivatives have demonstrated similar inhibitory properties in vivo, where their administration to diabetic rodent models resulted in improved oral glucose tolerance and reductions in fasting blood glucose. These findings suggest a predominantly intestinal mechanism that may contribute to postprandial glycemic modulation [5,7,8,67,68]. The principal mechanisms involved in glycemic modulation by *G. lucidum* are summarized in Figure 3.

### 4.2. Insulin Signaling and GLUT4 Translocation

Water-soluble polysaccharides derived from *G. lucidum* have been reported in experimental models to influence two complementary but not necessarily directly sequential signaling routes involved in glucose uptake and metabolic regulation. The IRS1–PI3K–Akt pathway is primarily associated with insulin-dependent GLUT4 translocation in skeletal muscle and adipose tissue, whereas AMPK functions as an energy-sensing pathway that can enhance glucose uptake and fatty acid oxidation through insulin-independent or partially insulin-independent mechanisms. Therefore, current evidence should be interpreted as supporting parallel or convergent modulation of insulin signaling and energy-sensing pathways rather than a confirmed direct activation of IRS1–PI3K–Akt by AMPK in the context of *G. lucidum* treatment [1,9,58,69,70].

The principal signaling pathways activated by water-soluble GLPs include the IRS1–PI3K–Akt axis and the AMPK pathway. Activation of IRS1–PI3K–Akt signaling supports insulin-dependent glucose uptake by promoting GLUT4 translocation to the cell membrane, particularly in skeletal muscle and adipose tissue [10,11,12,58]. AMPK activation provides an insulin-independent metabolic route that enhances glucose uptake, stimulates fatty acid oxidation, improves mitochondrial metabolic flexibility, and suppresses anabolic processes that contribute to insulin resistance [9,13,59,71,72]. These pathways are therefore complementary: PI3K–Akt signaling improves insulin responsiveness, whereas AMPK activation supports broader energy-sensing and metabolic adaptation [9,58].

AMPK is considered a key therapeutic target in type 2 diabetes because it functions as a cellular energy sensor that coordinates glucose and lipid metabolism [59,73]. When activated, AMPK enhances glucose uptake, promotes fatty acid oxidation, inhibits lipogenesis, reduces hepatic glucose production, and improves insulin sensitivity. These actions overlap with several clinically relevant metabolic goals in type 2 diabetes, including reduction in insulin resistance, improvement in hepatic glucose handling, and attenuation of lipid accumulation in insulin-sensitive tissues [73]. Therefore, AMPK activation by Ganoderma polysaccharide-rich preparations provides a mechanistic explanation for several preclinical metabolic effects, although this mechanism still requires stronger confirmation in human studies [9,59].

In parallel, activation of AMP-activated protein kinase is frequently observed following treatment with *G. lucidum* polysaccharide-rich extracts. This effect is consistent with improved insulin sensitivity, increased fatty acid oxidation, and greater metabolic flexibility. In diet-induced and obesity-associated models of insulin resistance, such signaling changes are accompanied by attenuation of weight gain and normalization of key metabolic parameters. Together, these observations align *G. lucidum* polysaccharides with AMP-linked mechanisms commonly associated with botanical agents exhibiting antidiabetic potential [9,59].

### 4.3. Regulation of Hepatic Glucose Output

The liver represents a central site of glucose homeostasis, and multiple lines of evidence indicate that *G. lucidum* polysaccharides modulate hepatic glucose metabolism. In hepatocyte cultures and diabetic rodent models, polysaccharide treatment is associated with reduced expression of gluconeogenic enzymes, including phosphoenolpyruvate carboxykinase and glucose-6-phosphatase, alongside activation of glycolytic and oxidative metabolic pathways. These coordinated effects contribute to lower fasting glucose levels and improvements in insulin resistance indices such as HOMA-IR [10,12,13,74,75].

The main gluconeogenic enzymes reported to be downregulated or inhibited following GLP treatment are phosphoenolpyruvate carboxykinase and glucose-6-phosphatase. Phosphoenolpyruvate carboxykinase catalyzes a rate-limiting step in gluconeogenesis, whereas glucose-6-phosphatase catalyzes the final step of hepatic glucose release into the bloodstream. Suppression of these enzymes reduces endogenous hepatic glucose production, which is particularly relevant for fasting hyperglycemia in type 2 diabetes. In diabetic animal models, reduced expression or activity of these enzymes has been associated with lower fasting glucose and improved insulin resistance indices [10,12,13,60].

In addition to polysaccharides, the well-characterized proteoglycan fraction FYGL exerts complementary actions on hepatic metabolism. FYGL has been shown to improve insulin sensitivity, enhance insulin secretion, and suppress hepatic glucose production in both cellular and animal models. Transcriptomic and metabolomic analyses further support these findings, highlighting FYGL as a multifunctional contributor to the regulation of hepatic glucose output [11,13].

### 4.4. β-Cell Protection and Insulin Secretion

Beyond peripheral insulin sensitivity, *G. lucidum* polysaccharides exert direct protective effects on pancreatic β-cells. Experimental data indicate that these compounds mitigate oxidative and endoplasmic reticulum stress within the islets of Langerhans, partly through downregulation of proapoptotic mediators such as Bax, caspase-3, and inducible nitric oxide synthase and upregulation of β-cell-associated and antiapoptotic factors, including PDX1 and Bcl2 [1,58].

To avoid oversimplification, β-cell protection should be interpreted as a multifactorial process rather than as a single pathway effect. In experimental diabetic models, Ganoderma-derived polysaccharides may reduce oxidative injury, attenuate inflammatory cytokine signaling, limit mitochondrial apoptosis, and preserve β-cell identity-associated transcriptional activity. Reduced expression of proapoptotic mediators such as Bax and caspase-3, together with increased or preserved expression of antiapoptotic and β-cell function-related markers such as Bcl2 and PDX1, may contribute to improved β-cell survival and maintenance of insulin secretory capacity. However, these effects remain primarily preclinical and require confirmation in human metabolic studies [11,13,16,58].

Changes in the expression of these genes are biologically important for β-cell survival. Bax promotes mitochondrial apoptosis, and its upregulation is associated with β-cell loss under oxidative or inflammatory stress. Bcl2 has the opposite function, stabilizing mitochondrial integrity and limiting apoptosis. Therefore, a lower Bax/Bcl2 ratio favors β-cell survival. PDX1 is a key transcription factor required for β-cell identity, insulin gene transcription, and maintenance of glucose-responsive insulin secretion. Preservation or upregulation of PDX1 therefore supports functional β-cell integrity, whereas loss of PDX1 is associated with β-cell dedifferentiation and impaired insulin production [11,13,16,58,76].

Ganoderma-derived polysaccharides may protect pancreatic β-cells through several convergent mechanisms. First, they reduce oxidative stress, which is particularly important because β-cells have relatively limited intrinsic antioxidant capacity and are highly vulnerable to reactive oxygen species. Second, they attenuate inflammatory signaling, thereby decreasing cytokine-mediated β-cell injury. Third, they modulate apoptosis-related pathways by reducing proapoptotic mediators and supporting antiapoptotic or β-cell identity-associated factors. In experimental diabetic models, these effects are associated with preservation of islet architecture, maintenance of insulin content, and improved insulin secretory capacity [11,13,16,58].

In streptozotocin-induced diabetic models, *G. lucidum* polysaccharides have been associated with improved glycemic control, modulation of apoptosis-related markers, and preservation or recovery of pancreatic β-cell function. Proteoglycan-rich preparations have also been reported to support pancreatic islet recovery in type 2 diabetic rats. These findings suggest that β-cell protection may contribute to the antihyperglycemic effects observed in diabetic animal models, although this evidence remains preclinical and should not be interpreted as proof of clinical efficacy in humans [11,16].

### 4.5. Anti-Inflammatory and Antioxidant Contributions

Chronic low-grade inflammation and oxidative stress are central contributors to insulin resistance, β-cell dysfunction, endothelial injury, hepatic steatosis, and progression of metabolic syndrome. In this context, the anti-inflammatory and antioxidant properties of *G. lucidum* may indirectly support glycemic regulation by reducing cellular stress pathways that impair insulin action and glucose homeostasis [17,21].

In vitro and animal studies indicate that polysaccharide-rich and triterpenoid-rich Ganoderma fractions can attenuate inflammatory signaling through modulation of NF-κB, MAPK, and related cytokine pathways [17,64,77,78,79]. These effects are commonly associated with reduced expression of pro-inflammatory mediators such as TNF-α, IL-6, IL-1β, iNOS, and COX-2 in metabolically relevant tissues, including liver, adipose tissue, pancreas, and skeletal muscle [13,17,77,78]. By reducing inflammatory signaling, Ganoderma-derived compounds may help preserve insulin receptor signaling and limit inflammation-induced impairment of IRS1–PI3K–Akt activity [13,21,64,80].

The antioxidant contribution is similarly relevant. Experimental models have shown that Ganoderma preparations may increase endogenous antioxidant defenses, including superoxide dismutase, catalase, glutathione peroxidase, and reduced glutathione while decreasing lipid peroxidation markers such as malondialdehyde [17,77,78]. These effects may protect pancreatic β-cells, which are particularly vulnerable to oxidative stress because of their relatively limited antioxidant capacity [13,58,81]. Preservation of β-cell integrity may contribute to improved insulin secretion in diabetic animal models [11,13,16].

At the hepatic level, antioxidant and anti-inflammatory effects may also reduce steatosis-associated metabolic dysfunction by limiting oxidative injury, suppressing inflammatory activation, and supporting AMPK-related metabolic adaptation. In the intestine, reduced oxidative and inflammatory stress may contribute to improved epithelial barrier function and lower metabolic endotoxemia, thereby linking antioxidant activity with microbiota-mediated metabolic effects [17,63,64,78,81].

Nevertheless, most of these mechanisms have been demonstrated in cellular systems or animal models. Therefore, the anti-inflammatory and antioxidant actions of *G. lucidum* should be interpreted as mechanistically plausible contributors to improved metabolic regulation rather than as independently confirmed clinical mechanisms for glycemic control in humans [77,82,83,84].

The simultaneous inhibition of oxidative and inflammatory stress is particularly important for preserving β-cell function because these two processes amplify each other. Oxidative stress can activate inflammatory pathways such as NF-κB, while inflammatory cytokines can increase reactive oxygen species production and promote mitochondrial dysfunction. In β-cells, this combined stress accelerates apoptosis, impairs insulin gene expression, and reduces glucose-stimulated insulin secretion. Therefore, Ganoderma-derived compounds that attenuate both oxidative damage and inflammatory signaling may provide broader cytoprotection than compounds acting on only one of these processes. However, this interpretation is based mainly on preclinical evidence and should be confirmed in human metabolic studies [13,17,58,64,77,78,85].

### 4.6. Modulation of the Gut Microbiota

As fermentable macromolecules, *G. lucidum* polysaccharides also exert metabolic effects through interactions with the gut microbiota. Preclinical studies indicate that these polysaccharides promote the expansion of short-chain fatty acid-producing bacterial taxa, enhance intestinal barrier integrity, and influence bile acid metabolism. Such microbiota-driven changes have been linked to reduced systemic inflammation, improved insulin sensitivity, and greater metabolic flexibility [14,15,18,63].

Recent integrative analyses emphasize that a substantial proportion of the metabolic benefits attributed to *G. lucidum* may arise from these microbiota-mediated mechanisms. Importantly, these effects appear to operate alongside direct host-targeted actions, including AMPK activation and insulin sensitization, reinforcing the concept of *G. lucidum* as a multilevel modulator of metabolic homeostasis [18,63].

The microbiota-related contribution should also be interpreted as a multistep mechanism. Fermentable Ganoderma polysaccharides may alter microbial community structure, favor short-chain fatty acid-producing taxa, and support epithelial barrier integrity. These changes may reduce metabolic endotoxemia and systemic inflammatory signaling, which are both relevant to insulin resistance and hepatic metabolic dysfunction. Thus, microbiota modulation may interact with hepatic glucose output, peripheral insulin sensitivity, and β-cell stress. Nevertheless, most of this evidence derives from animal studies, and causal confirmation in humans remains limited [14,15,18,63].

### 4.7. Interaction Between Intestinal and Systemic Mechanisms

The glycemic effects attributed to *G. lucidum* are best understood as the result of interactions between intestinal and systemic mechanisms rather than as a single-target action. At the intestinal level, lanostane-type triterpenoids may inhibit α-glucosidase and α-amylase, slow carbohydrate digestion, and reduce the rate of glucose absorption [5,7,8]. In parallel, fermentable polysaccharides may modulate gut microbiota composition, increase short-chain fatty acid production, improve epithelial barrier integrity, and reduce metabolic endotoxemia [14,15,18,63]. These intestinal effects can influence systemic metabolism by lowering postprandial glycemic load, reducing inflammatory signaling, and improving insulin sensitivity [18,63].

Systemically, GLPs and proteoglycan fractions may activate AMPK and IRS1–PI3K–Akt signaling in insulin-sensitive tissues, promote GLUT4-mediated glucose uptake [9,10,11,12,58], suppress hepatic gluconeogenesis, and protect pancreatic β-cells from oxidative and inflammatory injury [11,13,16,58]. The intestinal and systemic mechanisms are therefore interconnected: lower intestinal glucose absorption reduces postprandial stress on β-cells, microbiota-derived metabolites may support insulin sensitivity and hepatic metabolic regulation, and reduced systemic inflammation may improve both peripheral insulin signaling and β-cell survival [18,63]. This integrated gut–liver–pancreas–peripheral tissue axis provides a mechanistic framework for interpreting the preclinical glycemic effects of *G. lucidum*.

## 5. Evidence from Preclinical and Clinical Studies

### 5.1. Preclinical Evidence

In chemically and diet-induced models of type 2 diabetes mellitus, extracts of *G. lucidum*, particularly water-soluble polysaccharides (GLPs) and lanostane-type triterpenoids, have demonstrated antihyperglycemic effects [20]. GLP-enriched extracts have been reported to reduce fasting glucose and improve oral glucose tolerance and insulin sensitivity in diabetic rodents [10,11,12,13]. Other studies have described normalization of lipid metabolism, reduction in hepatic steatosis, and preservation of pancreatic islet and liver architecture [13,16,60].

Mechanistically, these effects have been attributed to modulation of IRS1–PI3K–Akt and AMPK signaling, increased GLUT4 expression or translocation, and improved peripheral glucose uptake [9,10,11,12]. Suppression of hepatic gluconeogenesis has also been linked to reduced expression or activity of phosphoenolpyruvate carboxykinase and glucose-6-phosphatase [10,12,13,60]. Highly purified fractions such as GLPF31 and the FYGL proteoglycan have been associated with improved glycemic parameters and reduced gluconeogenic gene expression in vivo (Table 3) [12,13].

In parallel, lanostane-type triterpenoids provide a complementary intestinal mechanism. Ganoderol B and other ganoderic or ganoderenic acid derivatives inhibit α-glucosidase and, in some experimental settings, α-amylase [5,7,8]. This mechanism may delay carbohydrate hydrolysis and attenuate postprandial increases in blood glucose, an effect conceptually comparable to acarbose in preclinical models [7,8,67]. Moreover, the FYGL proteoglycan has been associated with β-cell protective and insulinotropic effects, including improved insulin secretion, facilitated peripheral glucose uptake, reduced hepatic glucose production, and attenuation of oxidative stress [11,13]. Full syntheses from 2023 to 2025 agree on this multi-target pharmacological profile, where the relevance of incorporating both the pharmacological standardization of chemicals (GLP and triterpenoids) and the functional validation of the pharmacological target in vivo have been emphasized [2].

Therefore, the preclinical evidence supports mechanistic plausibility and potential antihyperglycemic activity, but these findings originate primarily from cellular systems and rodent models and should not be generalized as proven glycemic benefit in humans [5,7,9,10,11,12,13,16,60].

### 5.2. Clinical Studies

Human clinical evidence remains limited, heterogeneous, and overall inconclusive. The most methodologically rigorous human study to date was a double-blind, randomized, placebo-controlled trial involving 84 adults with type 2 diabetes mellitus and metabolic syndrome. Participants received *G. lucidum*, *G. lucidum* combined with *Cordyceps sinensis*, or a placebo for 16 weeks. No significant differences were observed between intervention and placebo groups in primary glycemic outcomes, including fasting glucose, HbA1c, and HOMA-IR, although the interventions were generally well tolerated (Table 4) [84].

Earlier small-scale studies, particularly those using spore powders or spore-derived preparations, reported favorable changes in postprandial glucose and lipid parameters. However, these findings should be interpreted cautiously because many of these studies were limited by inadequate randomization, incomplete blinding, short follow-up, and insufficient chemical standardization of the fungal preparation [82,83,86,87].

These mixed results are repeated in more recent comprehensive reviews and evidence summaries, which confirm the need for longer-duration, properly powered randomized clinical studies using genuine species and the method of dual chemical standardization, including the quantification of both GLP and the defined triterpenoid fingerprint, to properly assess efficacy for human metabolic disorders [82,83,87,88,89].

To improve readability and evidence traceability, the preclinical and clinical evidence tables were reformatted and expanded. References were added within the tables, repetitive study-type labels were removed where unnecessary, and the evidence was organized according to model, preparation, dose, endpoints, key findings, and interpretation.

**Table 3:** Preclinical evidence on the potential hypoglycemic effects of *G. lucidum*-derived preparations.

Study Type	Model	Compound/Source	Dose and Duration	Main Endpoints	Key Findings
STZ-induced diabetic rodents [10,16,60,90]	Streptozotocin (STZ)-induced diabetic rodents	GLP-rich aqueous fruiting body extract	Study-specific dose; variable duration	Fasting glucose, insulin, oxidative stress, tissue changes	Reduced fasting glucose and improved pancreatic/hepatic parameters.
Diet-induced obese mice [9,19]	Diet-induced obese or insulin-resistant mice	*G. lucidum* extract or polysaccharide-rich fraction	Study-specific dose; variable duration	Insulin resistance, AMPK, lipid metabolism	Improved insulin sensitivity and metabolic markers, often linked to AMPK activation.
db/db or T2DM rodent models [11,13]	db/db or T2DM rodent models	FYGL proteoglycan fraction	Study-specific dose; variable duration	Fasting glucose, insulin, hepatic glucose output, β-cell function	Improved glycemic control and insulin-related outcomes.
In vitro enzyme assay [7]	In vitro enzyme assays	Lanostane triterpenoids, ganoderol B, ganoderic acid derivatives	Concentration-dependent	α-glucosidase/α-amylase inhibition	Inhibited carbohydrate-digesting enzymes in vitro.
Cell culture model [9,10,11,12]	Cell culture models	GLPs or purified polysaccharides	Concentration-dependent	GLUT4, PI3K-Akt, AMPK, oxidative stress	Modulated insulin and energy-sensing pathways.
Gut microbiota animal model [14,15,18]	Gut microbiota animal models	GLP-rich preparations	Study-specific dose; variable duration	Microbiota, SCFAs (short-chain fatty acids), barrier function, inflammation	Altered microbiota and reduced inflammation-related metabolic dysfunction.

**Table 4:** Human clinical and review evidence on *G. lucidum* and metabolic regulation.

Evidence Type	Population/Focus	Intervention/Material	Duration/Dose	Main Outcomes	Main Conclusion	Limitations
Randomized controlled trial [84]	Adults with T2DM and metabolic syndrome	*G. lucidum*, alone or with *Cordyceps sinensis*	16 weeks; reported dose	Fasting glucose, HbA1c, HOMA-IR	No significant improvement versus placebo.	Moderate sample size, short duration, incomplete standardization.
Open-label/uncontrolled study [82,83,86]	Adults receiving spore-derived products	Spore powder or related preparation	Study-specific	Glucose levels, lipid profile	Some favorable metabolic changes were reported.	No placebo control, limited blinding, short follow-up.
Review evidence [2,21,82,83,88,89]	Ganoderma materials and metabolic outcomes	Mixed preparations	Not applicable	Mechanisms, translational evidence	Mechanistic plausibility exists, but clinical efficacy remains uncertain.	Reviews cannot replace primary clinical data.

### 5.3. Risk of Bias and Methodological Considerations in Clinical Evidence

The largest double-blind, randomized, placebo-controlled trial (n = 84) evaluating *G. lucidum* supplementation in individuals with type 2 diabetes mellitus and metabolic syn-drome did not demonstrate statistically significant improvements in primary glycemic endpoints after 16 weeks of intervention [84]. These findings represent the most methodologically robust clinical data currently available.

A qualitative risk-of-bias appraisal was therefore added for the available clinical evidence. For the largest randomized controlled trial, the overall risk of bias was considered lower than that of earlier intervention studies because the study used a randomized, double-blind, placebo-controlled design. These features reduce selection, performance, and detection bias. Nevertheless, several limitations remain relevant. The trial had a moderate sample size, a relatively short intervention period for detecting sustained changes in HbA1c, and the incomplete reporting of chemical fingerprinting and quantitative standardization of the administered Ganoderma preparation. These factors may introduce indirectness and limit reproducibility, even if the internal validity of the trial design is comparatively strong [84,88,89].

However, several design-related factors warrant careful consideration when interpreting these results. First, the intervention duration may have been insufficient to detect sustained changes in HbA1c, particularly in participants receiving stable background antidiabetic therapy. Second, the detailed chemical standardization and quantitative fingerprinting of the administered extract were not comprehensively reported, limiting reproducibility and cross-study comparability. Third, baseline metabolic control, heterogeneity in concomitant medication use, and potential ceiling effects may have reduced the likelihood of detecting additive metabolic benefits. Although the trial was appropriately randomized and placebo-controlled, the overall sample size may have limited statistical power for secondary endpoints [84,88,89].

Smaller open-label or short-duration interventional studies reporting favorable metabolic outcomes are constrained by the absence of placebo control, limited blinding procedures, short follow-up, and insufficient characterization of the fungal material. These limitations increase susceptibility to performance, detection, and reporting bias and restrict the strength of causal inference [82,83,86,91].

Overall, the risk of bias across the human evidence base was judged to be moderate to high, mainly because of heterogeneity in study design, limited sample size, short follow-up, incomplete blinding in some studies, the insufficient reporting of randomization procedures, and the poor characterization of the fungal material. Consequently, positive findings from smaller uncontrolled or weakly controlled studies were interpreted cautiously and were not considered sufficient to establish clinical efficacy [82,83,84,86,88,89].

Taken together, the current clinical evidence should be interpreted as inconclusive rather than definitively negative. The observed heterogeneity likely reflects variability in study design, material authentication, extract standardization, and statistical power, rather than the absence of biological plausibility suggested by preclinical data. Additionally, the insufficient reporting of extract composition and species authentication across studies significantly limits reproducibility and comparability of findings.

## 6. Safety, Toxicity Considerations, and Clinical Context

Placebo-controlled human studies indicate that authenticated Ganoderma preparations are generally well tolerated, with overall adverse event rates comparable to placebo [84,92]. Reported symptoms are predominantly mild and transient, most commonly involving gastrointestinal or constitutional complaints [84,86,92]. However, there are several signal-level safety considerations from a clinical and translational perspective.

From a toxicity perspective, *G. lucidum* preparations are generally regarded as having low acute toxicity when used at conventional oral doses [2,84,92]. However, toxicity assessment is complicated by major variation in species identity, tissue source, extraction method, concentration, and product quality [2,88]. Preclinical toxicity studies have usually reported a wide safety margin for aqueous polysaccharide-rich extracts, whereas concentrated spore powders, spore oils, ethanolic extracts, or poorly characterized commercial products may have different toxicological profiles [2,86,88]. Therefore, safety conclusions should not be generalized across all Ganoderma preparations. Attention should be given to hepatic adverse events [93,94], bleeding-related concerns, potential interactions with antidiabetic or anticoagulant drugs [84,86,92], and contamination risks involving heavy metals, pesticides, microbial contaminants, or undeclared adulterants [2,88]. In clinical translation, toxicity evaluation should include product authentication, contaminant testing, dose justification, adverse event monitoring, and when prolonged administration is planned, periodic assessment of liver function and coagulation-related parameters [88,93,94,95,96].

Experimental systems have shown that some water-soluble Ganoderma extracts inhibit platelet aggregation in vitro and ex vivo [95,96]. However, these findings have not been consistently confirmed in controlled clinical studies. Notably, a double-blind study in healthy volunteers did not find any clinically relevant effects on global hemostasis after short-term supplementation. However, when taken together, caution is warranted when using Ganoderma products with anticoagulant or antiplatelet therapies, and perioperative discontinuation remains a prudent precaution [92,95,96].

There are rare reports of hepatocellular injury temporally associated with the use of Ganoderma, which ranged from asymptomatic aminotransferase elevation to severe hepatitis. Although it is not possible to establish causality, with resolution usually occurring after discontinuation, these reports point to an idiosyncratic risk profile. Liver function monitoring is thus indicated during prolonged or high-dose administration, especially with spore-derived or concentrated formulations (Table 5) [93,94].

Given the glucose-lowering activity reported in preclinical and early clinical studies, additive hypoglycemic effects are possible when Ganoderma is used concurrently with insulin or insulin secretagogues. While clinically significant hypoglycemia seems rare, appropriate glucose monitoring and conventional dose adjustment are suggested [84].

Potential interactions with commonly used antidiabetic drugs should also be considered. With metformin, a pharmacodynamic overlap is plausible because both metformin and some Ganoderma polysaccharide-rich preparations are associated with AMPK activation and reduced hepatic glucose output in experimental models [9,73]. Although this does not necessarily indicate a harmful interaction, it may theoretically enhance glucose-lowering effects or complicate attribution of metabolic benefit in clinical studies [84,86]. When *G. lucidum* is used together with insulin, sulfonylureas, meglitinides, GLP-1 receptor agonists, SGLT2 inhibitors, DPP-4 inhibitors, or metformin, glucose monitoring is advisable, particularly during initiation, dose escalation, or use of concentrated extracts [84,86]. At present, clinically significant interactions between Ganoderma preparations and metformin have not been well established in controlled human studies; therefore, caution is based mainly on mechanistic plausibility and limited clinical evidence [9,73,84].

Further considerations concern the immunomodulatory effects of Ganoderma, which might be clinically relevant in cases of immunosuppressive treatment or after organ transplantation. Furthermore, spore derivatives form a chemically different group, and sporadic observations of tumor marker interference, such as CA72-4, call for oncological caution [2].

Misidentification of species and variation in the source of tissue and its methods of extraction further complicate the interpretation of the safety data. Thus, safety assessment and clinical application must be preceded by DNA-based authentication conducted with rigor, dual chemical standardization of triterpenoid and polysaccharide levels, and verification of compliance with pharmacopeial requirements for contaminants [25,34,88].

**Table 5:** Common adverse events and clinically relevant interactions with Ganoderma products.

Category	Typical Observations in Clinical or Observational Reports	Clinical Relevance	Recommended Actions
Gastrointestinal and constitutional adverse events	Dry mouth, nausea, dyspepsia, dizziness, insomnia, and headache, generally reported as mild and self-limiting	Incidence typically comparable to placebo and dependent on dose and formulation	Provide reassurance; consider dose reduction or formulation change if symptoms persist [84,86,92].
Bleeding and coagulation	In vitro and ex vivo evidence of platelet inhibition; isolated reports of perioperative bleeding	Potential increased bleeding risk when combined with anticoagulants or antiplatelet agents; randomized trials in healthy volunteers did not demonstrate global impairment of hemostasis	Avoid high-dose preparations in patients receiving anticoagulants or antiplatelet therapy unless supervised; discontinue use 1–2 weeks prior to surgery; monitor INR and clinical signs of bleeding [95,96].
Hepatotoxicity	Case reports ranging from mild elevations in alanine and aspartate aminotransferases to severe hepatitis, with fulminant liver failure reported rarely	Likely idiosyncratic reactions: risk may vary depending on product composition and tissue source	Perform baseline and periodic liver function testing during long-term or high-dose use; discontinue supplementation if liver enzymes increase or hepatic symptoms develop [93,94].
Glycemic interactions	Additive glucose-lowering effects when used concomitantly with antidiabetic medications	Symptomatic hypoglycemia reported infrequently	Educate patients regarding hypoglycemia symptoms; monitor blood glucose levels and adjust antidiabetic therapy as clinically indicated [84,86].
Immunomodulatory effects	Theoretical interaction with immunosuppressive therapies based on immunomodulatory properties	Clinical significance remains uncertain	Use with caution in transplant recipients or patients with autoimmune diseases; coordinate management with the treating clinical team [97,98,99].
Tumor marker interference (CA72-4)	Elevated CA72-4 levels reported in association with spore powder consumption	Potential confounding of oncological monitoring	Inform oncology teams of supplement use; interpret tumor marker results with caution [2].

### Regulatory Status and Commercial Use

The regulatory status of *G. lucidum* products varies according to jurisdiction, product type, intended use, and the specific fungal material used. In the United States, Reishi/Ganoderma products are commonly marketed as dietary supplements, but disease-treatment claims, including claims to treat diabetes or lower blood glucose as a therapeutic indication, may cause a product to be regarded as an unapproved new drug. Importantly, the presence of Ganoderma-derived ingredients in supplements does not mean that all preparations are generally recognized as safe for all uses. One specific FDA GRAS notice, GRN 413, concerns β-glucans derived from *G. lucidum* mycelium for defined uses in selected conventional food categories; this should not be generalized to all Ganoderma fruiting body extracts, spore oils, or concentrated supplements [100,101,102].

In the European Union, regulatory interpretation also depends on the form of the material and its history of consumption. Fruiting body preparations with a documented history of use may be treated differently from mycelium-derived powders, concentrated extracts, or novel processing forms, which may require assessment under the Novel Food framework. Therefore, regulatory status should not be described simply at the species level but rather at the level of the specific preparation, extraction method, intended use, dose, and target population [103].

From a translational perspective, these regulatory distinctions are important because many Ganoderma products are sold as foods, food supplements, or nutraceuticals rather than as approved antidiabetic therapies. Consequently, clinical claims should remain conservative unless supported by adequately powered human trials, standardized preparations, and jurisdiction-specific regulatory approval. Future studies should report the regulatory category of the tested product, its authentication method, contaminant testing, and compliance with local requirements for food supplements or novel foods [101,102,103].

## 7. Other Metabolically Relevant Activities

Although this review focuses on glycemic regulation, *G. lucidum* exhibits several pleiotropic metabolic effects that indirectly support glucose homeostasis, particularly within the metabolic syndrome spectrum. These broader metabolic effects further support the relevance of *G. lucidum* as a functional bioactive candidate within the context of metabolic syndrome and related non-communicable diseases [2,63,89].

Hypolipidemic activity has been consistently observed, with GLP-rich and mixed extracts reducing triglycerides, total cholesterol, and LDL-C while sometimes elevating HDL-C. These changes are mechanistically consistent with AMPK activation, enhanced fatty acid oxidation, and suppression of lipogenic pathways such as SREBP1c and FASN [20,64,86].

Hepatoprotective effects are also prominent: antioxidant and anti-inflammatory activities mitigate steatosis, lower ALT and AST levels, and improve hepatic histology in NAFLD-like models, actions that overlap mechanistically with AMPK upregulation and NF-κB inhibition [64].

Additionally, antioxidant defense enhancement through the upregulation of SOD, CAT, and GSH-Px and the suppression of lipid peroxidation reduce oxidative stress, a major contributor to insulin resistance and β-cell dysfunction [17,64,78].

From a translational perspective, these metabolic benefits are desirable yet mechanistically diverse across chemical classes, GLPs, triterpenoids, and spore-derived lipids. Future clinical trials should therefore stratify participants by metabolic endpoints (e.g., lipids, hepatic fat, and HOMA-IR) and employ dual chemical standardization to attribute efficacy to specific molecular constituents [21,83,88,89].

### Patent Landscape and Translational Relevance

The translational interest in Ganoderma-derived products is also reflected by patent activity related to polysaccharide fractions, spore-derived preparations, and multi-component traditional formulations. Several patent documents describe Ganoderma-based preparations intended for metabolic or cardiometabolic applications, including products claiming hypoglycemic, hypolipidemic, antioxidant, anti-obesity, or general metabolic-support effects. Examples include patents describing Ganoderma fruiting body polysaccharide preparations, polysaccharide fractions with anti-obesity properties, and traditional multi-component formulations containing Ganoderma materials for diabetes, hyperglycemia, hypertension, or hyperlipidemia [104,105].

However, patents should be interpreted differently from peer-reviewed experimental or clinical evidence. Patent claims generally indicate technological or commercial interest and may describe possible applications, formulations, extraction methods, or intended uses, but they do not necessarily provide independent confirmation of efficacy, standardized chemical characterization, or clinical benefit. For this reason, the patent landscape supports the translational relevance and commercial interest of Ganoderma-derived bioactives, but it cannot substitute rigorously designed pharmacological studies and adequately powered randomized clinical trials. Future translational development should therefore connect patentable formulations with transparent species authentication, reproducible extraction procedures, defined polysaccharide and triterpenoid marker profiles, toxicological evaluation, and clinically meaningful metabolic endpoints [104,105].

## 8. Discussion

### 8.1. Integration of Mechanistic Evidence

Accumulated experimental evidence, derived mainly from in vitro assays and animal models, supports a multi-target framework for the potential glycemic effects of *G. lucidum*. In cellular and rodent studies, water-soluble polysaccharides, including β-(1→3)/(1→6)-glucans, heteropolysaccharides, and proteoglycans, have been reported to improve insulin-related signaling through IRS1–PI3K–Akt pathways, GLUT4 translocation, and AMPK activation [1]. In diabetic rodent models, these effects are accompanied by suppression of hepatic gluconeogenesis, preservation of pancreatic β-cell function, and modulation of gut microbiota composition. In parallel, enzyme assays and preclinical studies suggest that lanostane-type triterpenoids act primarily within the intestinal compartment by inhibiting α-glucosidase and α-amylase, thereby moderating postprandial glucose excursions. Together, these findings provide a coherent biological rationale in preclinical systems, but they should not be interpreted as confirmed clinical efficacy in humans [1,89].

### 8.2. Translational Limitations in Human Studies

**Figure 4:**
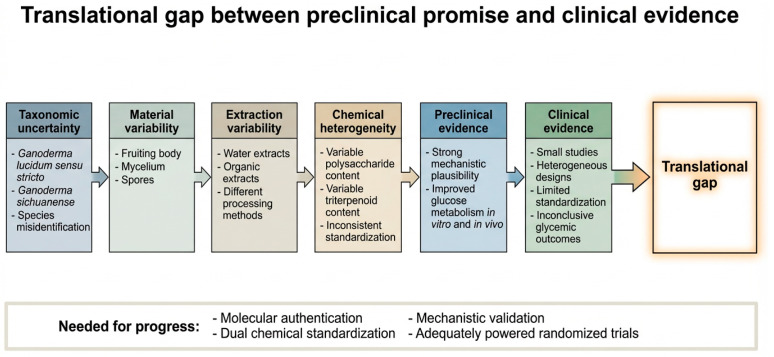
Conceptual framework illustrating the translational gap in *G. lucidum* research. Variability in taxonomic identification, tissue source, and extraction procedures contributes to substantial heterogeneity in chemical composition, particularly in polysaccharide and triterpenoid content. Although experimental studies consistently support mechanistic plausibility and beneficial metabolic effects in preclinical models, clinical studies remain limited by small sample sizes, heterogeneous designs, insufficient material characterization, and inconsistent glycemic outcomes. This translational gap underscores the need for rigorous molecular authentication, dual chemical standardization, mechanistic validation, and adequately powered randomized clinical trials.

Despite robust preclinical evidence, clinical outcomes remain inconsistent. At least one well-controlled randomized trial reported no significant benefit of *G. lucidum*, alone or combined with Cordyceps sinensis, over placebo for key glycemic endpoints [84]. This discrepancy likely reflects several sources of heterogeneity rather than an absence of biological activity. These include uncertainty in taxonomic identity, substantial differences among tissue sources, variability in extraction procedures and chemical composition, insufficient intervention duration to influence HbA1c, and limited statistical power due to small sample sizes and heterogeneous endpoints. Collectively, these factors complicate cross-study comparison and weaken mechanistic inference [21,88,89]. A conceptual overview of the translational gap between experimental evidence and clinical outcomes is presented in Figure 4.

### 8.3. Safety Considerations

Clinical studies to date indicate a generally favorable safety profile, with adverse events comparable to placebo. Nonetheless, caution is warranted in specific contexts, including potential bleeding risk with concomitant anticoagulant use, rare idiosyncratic hepatotoxic reactions, additive hypoglycemic effects when combined with antidiabetic therapy, and possible immunomodulatory interactions. Future trials should therefore incorporate pre-defined safety monitoring and thorough documentation of concomitant medications [21,84,92,93,94].

### 8.4. Remaining Knowledge Gaps

Important gaps remain, including a clearer definition of structure–activity relationships, an improved understanding of microbiota-mediated effects, and head-to-head comparisons of tissues and extraction strategies. Addressing these issues through well-designed, adequately powered trials will be necessary to clarify the therapeutic potential of *G. lucidum* in metabolic disease.

As a narrative synthesis, this review does not incorporate formal risk-of-bias scoring or quantitative meta-analysis, which reflects the methodological and chemical heterogeneity of the currently available literature [82,83,84,88,89].

A major source of inconsistency across studies is the lack of alignment between taxonomic identity, chemical composition, and biological activity. Future research should therefore adopt an integrated framework combining molecular authentication, dual chemical standardization, and mechanistic validation to improve translational reliability [5,21,25,34,43,88,89].

## 9. Conclusions

Available evidence supports a multifaceted role of *G. lucidum* in the modulation of glucose homeostasis. Polysaccharide fractions consistently enhance insulin sensitivity through AMPK and GLUT4-associated pathways, suppress hepatic gluconeogenesis, preserve pancreatic β-cell integrity, and interact with the gut–liver axis. Lanostane-type triterpenoids primarily influence postprandial glycemia via inhibition of α-glucosidase and α-amylase. These complementary mechanisms are well documented in preclinical models and provide a coherent biological rationale for metabolic effects [1,5,7,9,10,11,12,13,63,89].

Overall, the glycemic regulatory potential of *G. lucidum* appears to depend on complementary compound classes and mechanisms: polysaccharides and proteoglycans mainly influence systemic insulin signaling, hepatic glucose production, β-cell protection, and microbiota-mediated effects, whereas lanostane-type triterpenoids mainly contribute to intestinal carbohydrate enzyme inhibition and postprandial glucose modulation [1,5,7,9,11,12,13,63,89].

However, clinical translation remains uncertain. Human studies are limited by heterogeneity in species authentication, tissue source, extract composition, and insufficient chemical and functional standardization. In addition, short intervention durations and modest sample sizes have constrained the ability to detect sustained improvements in endpoints such as HbA1c.

Future research should prioritize rigorous species authentication, defined chemofunctional marker panels, and adequately powered clinical trials of sufficient duration aligned with mechanistic endpoints. From a translational perspective, *G. lucidum* should also be considered within the broader field of functional foods and bioactive natural products investigated for the prevention or attenuation of metabolic syndrome-related disorders.

In addition, future translational studies should consider phytochemical stability, oral bioavailability, extraction methodology, possible interactions with antidiabetic drugs, and the regulatory classification of the tested preparation. These factors are essential to distinguishing general nutraceutical use from clinically validated metabolic interventions [43,54,84,88,89,101,102,103].

At present, *G. lucidum* should be regarded as a mechanistically plausible but clinically unproven adjunct in metabolic regulation.

## Figures and Tables

**Table 1:** Major phytochemical classes of *Ganoderma lucidum* and their proposed relevance to glycemic regulation.

Constituent Class	Typical Source	Representative Markers	Principal Glycemic Mechanisms
GL polysaccharides/water-soluble polysaccharides	Fruiting body, mycelium	β-(1→3)/(1→6)-glucans; heteropolysaccharides	AMPK activation; improved insulin sensitivity; GLUT4 translocation; hepatic gluconeogenesis suppression; gut microbiota modulation
Proteoglycan fractions	Fruiting body aqueous extracts	FYGL; glycoprotein-rich fractions	Insulin sensitization; α-glucosidase inhibition; β-cell protection; reduced hepatic glucose output
Lanostane-type triterpenoids	Fruiting body organic extracts, spores	Ganoderic acids; ganoderenic acids; ganoderols; ganodermanols	α-Glucosidase and α-amylase inhibition; postprandial glucose modulation; antioxidant and anti-inflammatory support
Phenolic compounds	Fruiting body, mycelium, extracts	Phenolic acids; flavonoid-like compounds; antioxidant phenolics	Free-radical scavenging; reduction in oxidative stress; indirect β-cell and hepatic protection
Spore-derived lipid fractions	Broken or processed spores	Triterpenoid-rich oils; sterols; fatty acids	Antioxidant and anti-inflammatory metabolic support; possible glycemic modulation; activity depends strongly on processing

**Table 2:** Analytical standardization map for *G. lucidum* hypoglycemic constituents: quantitative markers, methods, and mechanism linkage.

Constituent Class	Typical Source	Representative Markers	Recommended Analytical Methods	Principal Glycemic Mechanisms
GL polysaccharides (GLPs)	Fruiting body, mycelium	β-(1→3)/(1→6)-glucans	Total carbohydrate assay; HPSEC-MALS; PMP-HPLC or GC/MS; FTIR/NMR	AMPK activation; GLUT4 translocation; hepatic gluconeogenesis suppression; β-cell protection; microbiota modulation
Proteoglycan fraction (FYGL)	Fruiting body (aqueous extract)	Glycoprotein rich fraction	Protein/carbohydrate quantification; HSPEC-MALS; SDS-PAGE; FTIR/NMR	Insulin sensitization; inhibition of hepatic glucose output; α-glucosidase inhibition (fraction dependent)
Lanostane triterpenoids	Fruiting body (organic extracts)	Ganoderic acids; Ganoderol B	HPLC-DAD or UPLC-DAD; LC-MS/MS	α-Glucosidase and α-amylase inhibition; postprandial glucose attenuation
Spore-derived preparations (oil or powder)	Broken/processed spores	Triterpene-rich lipid fractions	Sporoderm confirmation; LC-MS/MS; GC-MS; contaminant testing	Enzyme inhibition (fraction dependent); antioxidant and anti-inflammatory metabolic support

## Data Availability

No new data were generated in this study.

## Supplementary Materials

The following supporting information can be downloaded at: https://www.mdpi.com/article/10.3390/metabo16050334/s1, Table S1: Analytical and methodological standardization framework for *Ganoderma lucidum* hypoglycemic constituents.

## Abbreviations

The following abbreviations are used in this manuscript:

AMPK	AMP-activated protein kinase
FYGL	Fudan–Yueyang *Ganoderma lucidum* proteoglycan
GL	*Ganoderma lucidum*
GLPs	*Ganoderma lucidum* polysaccharides
GLUT4	Glucose transporter type 4
HbA1c	Glycated hemoglobin A1c
HOMA-IR	Homeostatic model assessment of insulin resistance
IRS1	Insulin receptor substrate 1
NAFLD	Non-alcoholic fatty liver disease
OGTT	Oral glucose tolerance test
PI3K	Phosphoinositide 3-kinase
T2DM	Type 2 diabetes mellitus
