# Supplementary material for: Ganoderma lucidum as a Functional Bioactive Candidate for Glycemic Regulation: Mechanisms, Preclinical Evidence, and Clinical Translation
Source: Metabolites. 2026 May 15;16(5):334. doi: 10.3390/metabo16050334 (PMC13208482; doi:10.3390/metabo16050334)
Supplement: Supplementary file 1 [file metabolites-16-00334-s001.zip › metabolites-4289431-supplementary.pdf]

Supplementary Materials

Table S1. Detailed analytical standardization map for *Ganoderma lucidum* hypoglycemic constituents.

Constituent group	Tissue / matrix	Key quantitative markers (minimum)	Core analytical methods (MDPI QA–fit)	Mechanistic linkage (Section 4)	Standardization & reporting essentials
**GL polysaccharides (GLPs)** β-(1→3)/(1→6)-glucans; heteropolysaccharides	Fruiting body (hot-water); mycelium (submerged/solid-state); spores (water-soluble)	Total carbohydrates (%); total β-glucans (soluble + insoluble); monosaccharide molar ratio (Glc, Gal, Man, Fuc, Xyl, Rha); Mw/Mn, dispersity	Phenol–sulfuric acid; enzymatic or aniline blue β-glucan assays; HPSEC–MALS; PMP–HPLC or alditol acetate GC–MS; FT-IR; $^{1}H/^{13}C$ NMR	4.2 Insulin signaling/GLUT4; 4.3 Hepatic glucose output; 4.4 β-cell protection; 4.5 Anti-inflammatory/antioxidant; 4.6 Gut microbiota	State tissue and extraction; report mean ± SD across batches; include MW profiles and chromatograms (SI); deposit voucher or culture ID
**Exo-/endo-β-glucans (bioreactor-derived)**	Mycelial broth or biomass	Yield (g/L); β-glucan %; β-(1→3)/(1→6) linkage ratio; viscosity	HPSEC–MALS; methylation analysis + GC–MS; rheometry	4.2 GLUT4/AMPK signaling; 4.6 Prebiotic effects	Report fermentation parameters (C/N source, pH, DO, temperature); define harvest time and its impact on MW/branching
**GL proteoglycan FYGL** (glycoprotein-rich fraction)	Fruiting body or mycelium (aqueous, protein-bound)	Protein %; carbohydrate %; glycan-to-protein ratio; peptide/protein fingerprint	BCA or Kjeldahl/Dumas; SDS-PAGE; LC–MS; HPLC/GC–MS monosaccharides; HPSEC–MALS	4.3 Hepatic gluconeogenesis; 4.3–4.4 Insulin secretion/β-cell function; 4.1 α-glucosidase inhibition (fraction-dependent)	Define isolation workflow; specify strain/source; include a bioactivity fingerprint (e.g., α-glucosidase IC_{50}, insulinotropic assay)
**Lanostane triterpenoids (panel)**ganoderic acids A, B, C_2; ganoderenic acids	Fruiting body (EtOH/EtOAc); lipid-enriched spores	Absolute/relative content of ≥5 marker acids; total lanostanes	HPLC/UPLC-DAD; LC–MS/MS; optional qNMR	4.1 α-glucosidase/α-amylase inhibition; supportive 4.5 anti-inflammatory effects	Report individual and total lanostanes (mg/g extract); compare with authenticated standards; include stability data
**Spore oil (triterpene-rich lipid)**	Broken or sporoderm-removed spores (oil)	Total triterpenes (GA-B equivalents); sterol/triterpene profile;	LC–MS/MS (lanostanes/sterols); GC–FID (fatty acids); AOCS oxidative indices	4.1 Enzyme inhibition (fraction-dependent); 4.5 Antioxidant effects	Declare sporoderm status; report oxidation control and limits; note potential

		peroxide and acid values			anticoagulant interactions
**Exemplar compounds**ganoderol B; ganodermanontriol	Fruiting body (organic extracts)	Purity; structural identity; α-glucosidase IC_{50}	Semi-prep HPLC; $^{1}H/^{13}C$ NMR, HSQC, HMBC; MS/MS	4.1 Competitive α-glucosidase inhibition	State enzyme source, substrate, buffer, temperature; report Ki and inhibition mode
**Functional bioassays (cross-cutting)**	Extracts/fractions matched above	α-Glucosidase/α-amylase IC_{50}; GLUT4 translocation; AMPK/Akt phosphorylation; PEPCK/G6Pase; GSIS; antioxidant capacity	Standardized enzyme assays; confocal GLUT4; Western blot/ELISA; qPCR/protein assays; INS-1/Min6 GSIS; validated antioxidant assays	Sections 4.1–4.5	Always pair chemistry with function; include positive controls (acarbose, metformin, insulin); predefine primary endpoints
**Constituent group**	**Tissue / matrix**	**Key quantitative markers (minimum)**	**Core analytical methods (MDPI QA–fit)**	**Mechanistic linkage (Section 4)**	**Standardization & reporting essentials**
**GL polysaccharides (GLPs)** β-(1→3)/(1→6)-glucans; heteropolysaccharides	Fruiting body (hot-water); mycelium (submerged/solid-state); spores (water-soluble)	Total carbohydrates (%); total β-glucans (soluble + insoluble); monosaccharide molar ratio (Glc, Gal, Man, Fuc, Xyl, Rha); Mw/Mn, dispersity	Phenol–sulfuric acid; enzymatic or aniline blue β-glucan assays; HPSEC–MALS; PMP–HPLC or alditol acetate GC–MS; FT-IR; $^{1}H/^{13}C$ NMR	4.2 Insulin signaling/GLUT4; 4.3 Hepatic glucose output; 4.4 β-cell protection; 4.5 Anti-inflammatory/antioxidant; 4.6 Gut microbiota	State tissue and extraction; report mean ± SD across batches; include MW profiles and chromatograms (SI); deposit voucher or culture ID
**Exo-/endo-β-glucans (bioreactor-derived)**	Mycelial broth or biomass	Yield (g/L); β-glucan %; β-(1→3)/(1→6) linkage ratio; viscosity	HPSEC–MALS; methylation analysis + GC–MS; rheometry	4.2 GLUT4/AMPK signaling; 4.6 Prebiotic effects	Report fermentation parameters (C/N source, pH, DO, temperature); define harvest time and its impact on MW/branching
